# Bile acid detoxifying enzymes limit susceptibility to liver fibrosis in female SHRSP5/Dmcr rats fed with a high-fat-cholesterol diet

**DOI:** 10.1371/journal.pone.0192863

**Published:** 2018-02-13

**Authors:** Husna Yetti, Hisao Naito, Yuan Yuan, Xiaofang Jia, Yumi Hayashi, Hazuki Tamada, Kazuya Kitamori, Katsumi Ikeda, Yukio Yamori, Tamie Nakajima

**Affiliations:** 1 Department of Occupational and Environmental Health, Nagoya University Graduate School of Medicine, Nagoya, Japan; 2 Department of Public Health, Fujita Health University School of Medicine, Toyoake, Japan; 3 College of Life and Health Sciences, Chubu University, Kasugai, Japan; 4 College of Human Life and Environment, Kinjo Gakuin University, Nagoya, Japan; 5 School of Pharmacy and Pharmaceutical Sciences, Mukogawa Women’s University, Nishinomiya, Japan; 6 Institute for World Health Development, Mukogawa Women’s University, Nishinomiya, Japan; Max Delbruck Centrum fur Molekulare Medizin Berlin Buch, GERMANY

## Abstract

During middle age, women are less susceptible to nonalcoholic steatohepatitis (NASH) than men. Thus, we investigated the underlying molecular mechanisms behind these sexual differences using an established rat model of NASH. Mature female and male stroke-prone spontaneously hypertensive 5/Dmcr rats were fed control or high-fat-cholesterol (HFC) diets for 2, 8, and 14 weeks. Although HFC-induced hepatic fibrosis was markedly less severe in females than in males, only minor gender differences were observed in expression levels of cytochrome P450 enzymes (CYP)7A1, CYP8B1 CYP27A1, and CYP7B1, and multidrug resistance-associated protein 3, and bile salt export pump, which are involved in fibrosis-related bile acid (BA) kinetics. However, the BA detoxification-related enzymes UDP-glucuronosyltransferase (UGT) and sulfotransferase (SULT) 2A1, and the nuclear receptors constitutive androstane receptor (CAR) and pregnane X receptor (PXR), were strongly suppressed in HFC-fed males, and were only slightly changed in HFC-diet fed females. Expression levels of the farnesoid X receptor and its small heterodimer partner were similarly regulated in a gender-dependent fashion following HFC feeding. Hence, the pronounced female resistance to HFC-induced liver damage likely reflects sustained expression of the nuclear receptors CAR and PXR and the BA detoxification enzymes UGT and SULT.

## Introduction

Nonalcoholic fatty liver disease (NAFLD) is the most common chronic liver disease in developed and developing countries [1–3]. Nonalcoholic steatohepatitis (NASH) is the progressive form of NAFLD, and leads to cirrhosis, hepatocellular carcinoma, and hepatic failure, and is a serious public health problem [4]. The prevalence of NASH/NAFLD varies with gender and age in humans, and in a study of 193 Japanese patients with biopsy-diagnosed NASH, male gender was more prevalent among patients of 30–40 years of age, whereas female gender was predominant among patients of > 50 years of age [5]. In accordance, a recent prospective study using ultrasound analyses and liver biopsies showed that NAFLD was more frequent in male than in female middle-aged patients [6].

Animal experiments using *Pten* knockout mice demonstrated that females have attenuated hepatic steatosis, inflammation, and carcinogenicity compared with male mice [7]. However, this model was based on modifications of genes that are involved in carcinogenesis. In contrast, female mice were reportedly more susceptible to NAFLD induced by 30% fructose [8], and methionine-choline-deficient diet (MCDD)-induced steatohepatitis was comparable in male and female mice [9]. Hence, although gender differences in the development of NAFLD/NASH have been investigated in several animal studies, contrasting conclusions are reported. Moreover, the mechanisms underlying gender-related differences in NAFLD/NASH remain poorly understood, warranting development of an appropriate animal model for evaluating gender differences in NASH/NAFLD and clarifying the related mechanisms.

Cholesterol contributed to NASH progression in humans [10, 11] and in animal models [12–14]. In hepatocytes, cholesterol is catabolized into bile acids (BAs) [15], which may cause hepatotoxicity and liver damage [16]. In addition, increasing BA levels were confirmed in livers from NASH patients [17] and in serum and liver samples from rats with NASH/NAFLD [18, 19]. In a previous study, we established a fibrotic steatohepatitis model by feeding male stroke-prone spontaneously hypertensive rats (SHRSP5/Dmcr) with a high-fat-cholesterol (HFC) diet for 8 weeks, and demonstrated histopathological resemblance to human NASH [13, 20]. We also showed that BAs and enzymes and promoters of BA kinetics play crucial roles in hepatic inflammation and fibrogenesis in this rat model [21–23]. Therefore, this model is likely appropriate for further investigations of the mechanisms behind gender differences in HFC-induced fibrotic steatohepatitis. Herein, we compared histopathological and molecular characteristics of fibrotic steatohepatitis between female and male HFC diet fed SHRSP5/Dmcr rats, and showed gender-specific responses of BA kinetics and nuclear receptor expression levels.

## Materials and methods

### Animal and diets

All experiments were approved by the Committee for Ethics of Animal Experiments at the Kinjo Gakuin University Animal Centre (Ethical approval code No. 10 and 27). Eighteen male and fifty-three female 10-week-old SHRSP5/Dmcr rats were generated as described previously [13] and were housed at 23°C–25°C with 55%–60% relative humidity and a 12-h light/12-h dark cycle. Animals were assigned to 6 groups for each gender (males, n = 6/group and females, n = 7–10/group). Subsequently, 3 treatment groups for each gender were fed an SP (Stroke-prone) diet as controls, and the remaining 3 groups were fed a HFC diet for 2, 8, or 14 weeks. Contents of control and HFC diets were described in detail previously [23]. After 18–20-h fasting, all rats were sacrificed under anesthesia using pentobarbital (70 mg/kg), and blood and liver samples were taken. Part of the samples were fixed in 4% buffered paraformaldehyde for histological examinations, and the remaining liver samples were immediately stored at −80°C for subsequent analysis. Serum was collected after centrifuging blood samples at 3,500× g for 10 min and was stored at −80°C until analysis. In order to align the estrous cycle, serum estradiol levels in female rats were measured by ASKA pharmaceutical Medical Co., LtD (Kawasaki, Japan). Liver and serum samples of the six male and six female rats with the lowest estradiol levels were selected from each group, and were used in subsequent experiments.

### Histopathology

Histopathological changes were investigated in formalin-fixed liver tissues (4-μm sections) using hematoxylin and eosin (H&E) staining, and necrotic areas were scored as described previously [23]. Modified Elastic Van Gieson (EVG) staining was performed using Sirius red to evaluate fibrosis areas. Specimens were examined under a DM750 microscope (Leica, Wetzlar, Germany). Fibrosis areas were evaluated using NIS-Elements software (Nikon instruments, Tokyo, Japan) as described previously [18].

### Biochemical assays of serum and liver extracts

Serum aspartate aminotransferase (AST), alanine aminotransferase (ALT), γ-glutamyl transpeptidase (GGT), triglyceride (TG), and total cholesterol (TC) levels were determined by SRL. Inc. (Tokyo, Japan). Serum TNF-α was measured using commercial kits (R&D Systems Inc., Minneapolis, MN). TG and TC levels in the livers were measured as described previously [13], and all experiments were repeated at least two times.

### UGT activity assays

UGT activity was determined using a UGT-GloTM Assay Kit (Promega, Madison, WI) according to the manufacturer’s instructions with 0.2 mg of hepatic microsomes and 50 μl of multienzyme substrates at 37°C for 30 min.

### Real-time quantitative polymerase chain reaction

Total RNA was isolated from whole livers using RNeasy Mini Kits (QIAGEN, Tokyo, Japan) and quantitative real-time polymerase chain reaction (PCR) analyses were performed as described previously [24] using an Applied Biosystems 7900HT Fast Real Time PCR System (Thermo Fisher Scientific, Waltham, MA) and the primers listed in S1 Table. In these experiments, mRNA expression levels were normalized to that of *glyceraldehyde 3-phosphate dehydrogenase* (*Gapdh*) using the ΔΔCT method.

### Western blot analysis

Frozen liver tissues were homogenized in buffer containing 0.25-M sucrose and 10-mM phosphate (pH 7.4). Nuclear fractions were extracted from parts of frozen liver samples using CelLytic^TM^ NuCLEAR^TM^ Extraction Kits (Sigma-Aldrich Japan, Tokyo, Japan). Samples were then subjected to 10% or 12.5% sodium dodecyl sulfate-polyacrylamide gel electrophoresis and proteins were transferred to polyvinylidene difluoride membranes as described previously [23]. Membranes were then incubated overnight at 4°C with primary polyclonal antibodies against alpha smooth muscle actin (α-SMA), cytochrome (CYP)7A1, CYP27A1 (Abcam plc, Cambridge, UK), constitutive androstane receptor (CAR; GeneTex, Inc., Irvine, CA), bile salt export pump (BSEP), CYP7B1, CYP8B1, farnesoid X receptor (FXR), pregnane X receptor (PXR), small heterodimer partner (SHP), sulfotransferase (SULT)2A1, transforming growth factor (TGF)-β1 (Santa Cruz Biotechnology, Santa Cruz, CA), or multidrug resistance-associated protein 3 (MRP3; Sigma-Aldrich Japan). GAPDH (Santa Cruz Biotechnology) and TATA binding protein (TBP; Abcam plc) were used as loading controls for homogenates and nuclear fractions, respectively. Protein signals were detected using ECL Western Blotting Detection Reagent (GE Healthcare, Buckinghamshire, UK).

### Statistical analysis

Data are expressed as means ± standard deviations (SD). Group differences at the same time points in males and females, and differences in fold changes following HFC *vs*. control diets between males and females at each time point were identified using Student’s *t*-test. Nonparametric data were normalized using logarithmic or square root transformations. All analyses were performed using Statistical Package for the Social Sciences program version 21, and differences were considered significant at probability (*P*) value < 0.05.

## Results

### Changes in body weight, liver weight, and liver/body weight ratios

HFC diet feeding decreased body weights compared to respective controls at all time points in male rats, but decreased significantly only at 2 weeks in females (Table 1). Moreover, relative to controls, fold changes in body weights of HFC-fed rats were significantly lower in males than in females at 8 and 14 weeks. In contrast, the HFC diet increased liver weights in both male and female rats, and when expressed relative to data from control diet-fed mice, fold changes were comparable between female and male rats at all time points. However, although the HFC diet increased liver weights relative to body weights in both males and females compared with the control diet, fold increases in female rats were lower than in males at 8 weeks. Thus, in response to HFC feeding, female rats exhibited a minor liver weight increase and body weight decrease than male rats.

**Table 1:** Body weight, liver weight, relative liver weight, levels of serum and liver lipids, serum liver function indices, and levels of cytokine.

Parameters	2 weeks				8 weeks				14 weeks			
	Male		Female		Male		Female		Male		Female	
	Control	HFC	Control	HFC	Control	HFC	Control	HFC	Control	HFC	Control	HFC
Body weight (g)	263 ± 19	242 ± 13* (0.9)	15 5 ± 10	145 ± 6* (0.9)	314 ± 20	268 ± 14* (0.9)	179 ± 6	186 ± 8 (1.04)#	336 ± 22	277 ± 19* (0.8)	196 ± 10	188 ± 15 (0.95)#
Liver weight (g)	7.3 ± 0.7	11.6 ± 3.8* (1.6)	5.7 ± 0.4	9.0 ± 0.5* (1.6)	8.3 ± 0.8	34.6 ± 3.8* (4.2)	6.1 ± 0.6	22.5 ± 1.1* (3.7)	9.3 ± 0.7	37.5 ± 4.9* (4)	6.1 ± 0.2	26.7 ± 2.7* (4.4)
Relative liver weight (%)	2.8 ± 0.1	4.8 ± 1.5* (1.7)	3.7 ± 0.1	6.2 ± 0.4* (1.7)	2.6 ± 0.1	12.9 ± 1.2* (4.9)	3.4 ± 0.3	12.1 ± 0.9* (3.6)#	2.8 ± 0.1	13.5 ± 1.3* (4.9)	3.1 ± 0.1	14.2 ± 0.8* (4.6)
Serum												
AST (IU/L)	121 ± 12	144 ± 11* (1.2)	136 ± 37	169 ± 35 (1.2)	105 ± 10	545 ± 209* (5.2)	122 ± 29	626 ± 233* (5.1)	118 ± 21	1121 ± 320* (9.5)	117 ± 26	874 ± 203* (7.5)
ALT (IU/L)	47 ± 3.5	94 ± 15* (2)	43 ± 5.2	76 ± 14* (1.8)	52 ± 4.1	220 ± 66* (4.3)	48 ± 15	213 ± 56* (4.5)	50 ± 5.5	387 ± 87* (7.7)	44 ± 5.5	462 ± 104* (10.5)
γ-GTP (IU/L)ǂ	1.5 ± 0	1.5 ± 0 (1.0)	1.5 ± 0	1.5 ± 0 (1.0)	1.5 ± 0	10.3 ± 2* (6.9)	1.5 ± 0	5.9 ± 5.5 (3.9)	1.5 ± 0	13.8 ± 2.9* (9.2)	1.5 ± 0	8.5 ± 7.1* (5.7)
TNF-α (pg/ml)ǂ	1.7 ± 1.8	7.2 ± 3.3* (4.2)	2.5 ± 0	2 ± 0.7 (0.8)#	1.3 ± 1	13 ± 3.3* (9.8)	2.5 ± 0	28 ± 6.3* (9.5)	3 ± 1.5	16 ± 6.2* (5.3)	2.5 ± 0	19 ± 9.5* (7.6)
TC (mg/dl)	51 ± 1.6	129 ± 52* (2.5)	65 ± 6	732 ± 196* (11.4)#	61 ± 5	266 ± 157* (4.3)	75 ± 7	882 ± 397* (11.8)#	69 ± 3	1593 ± 884*(23.2)	75 ± 6	1991 ± 904* (26.6)
TG (mg/dl)	36 ± 13	41 ± 5.4 (1.1)	18 ± 4.6	80 ± 24* (4.5)#	37 ± 9	29 ± 11 (0.76)	22 ± 6.7	122 ± 24* (5.7)#	42 ± 7.7	79 ± 63 (1.9)	18 ± 7.7	424 ± 292* (23)#
Estradiol (pg/ml)	ND	ND	12.3 ± 12.7	1.3 ± 1.5*	ND	ND	3.4 ± 3.8	0.8 ± 1.0	ND	ND	9.6 ± 12.2	1.3 ± 0.4
Liver												
Liver TC (mg/g liver)	2.0 ± 0.4	115 ± 23* (57)	2.7 ± 0.1	71 ± 14* (26)#	2.0 ± 2.0	128 ± 11* (64)	2.6 ± 0.1	127 ± 10* (49)#	1.9 ± 0.2	166 ± 30* (89)	2.3 ± 0.6	154 ± 27* (67)#
Liver TG (mg/g liver)	19 ± 3	56 ± 27* (2.9)	13 ± 4	36 ± 13* (2.8)	18 ± 5	36 ± 12* (2)	16 ± 4	34 ± 6* (2.1)	15 ± 3	28 ± 7* (1.9)	16 ± 3	25 ± 6* (1.5)

Data from male rats were previously presented [20, 21]. Values are expressed as means ± standard deviations and those in the parentheses are presented as mean fold changes in the HFC group with respect to that in the control group.

* P < 0.05 vs. control

# P < 0.05 vs. male fold change of the HFC-fed group at the same time point

n = 6/group.

Limits of detection were 3.0 IU/L for γ-GTP and 5 pg/ml for TNF-α. Values below the detection limit are presented as 1.5 IU/L and 2.5 pg/ml, respectively.

Abbreviations: HFC, high-fat-cholesterol; TC, total cholesterol; TG, triglyceride, AST, aspartate aminotransferase; ALT, alanine aminotransferase; γ-GTP, γ-glutamyl transpeptidase; TNF-α, tumor necrosis factor-α; ND, not determined.

### Gender differences in biochemical measurements of serum and liver

The present HFC diet significantly increased serum AST and ALT levels compared to respective controls in males and females at all time points except in females at 2 weeks (Table 1). However, no gender differences in AST or ALT increases were observed. Serum γ-GTP levels were only detected in serum from HFC diet-fed male and female rats at 8 and 14 weeks, respectively, and no gender differences were identified. HFC feeding increased pro-inflammatory TNF-α expression in both sexes except in females at 2 weeks, and these increases were significantly greater in males than in females at 2 weeks. HFC diet suppressed serum estradiol levels in females only at 2-week feeding.

The HFC diet did not increase serum TG levels in male rats but significantly increased serum TG levels in females at all time points. In contrast, HFC diet feeding significantly increased serum TC levels in male and female rats at all time points, although these increases were significantly greater in female rats than in male rats at 2 and 8 weeks.

In liver tissues, TG and TC levels were increased by HFC feeding in both male and female rats. However, whereas increases of TG levels in female rats were comparable to those in males, increases in liver TC levels were significantly lower in females than in males at all time points.

Average serum estradiol levels in the six female rats with the lowest estradiol levels in each group were significantly decreased by HFC feeding at 2 weeks, but not affected at 8 and 14 weeks.

### Gender differences in HFC-induced histopathological changes

**Fig 1:**
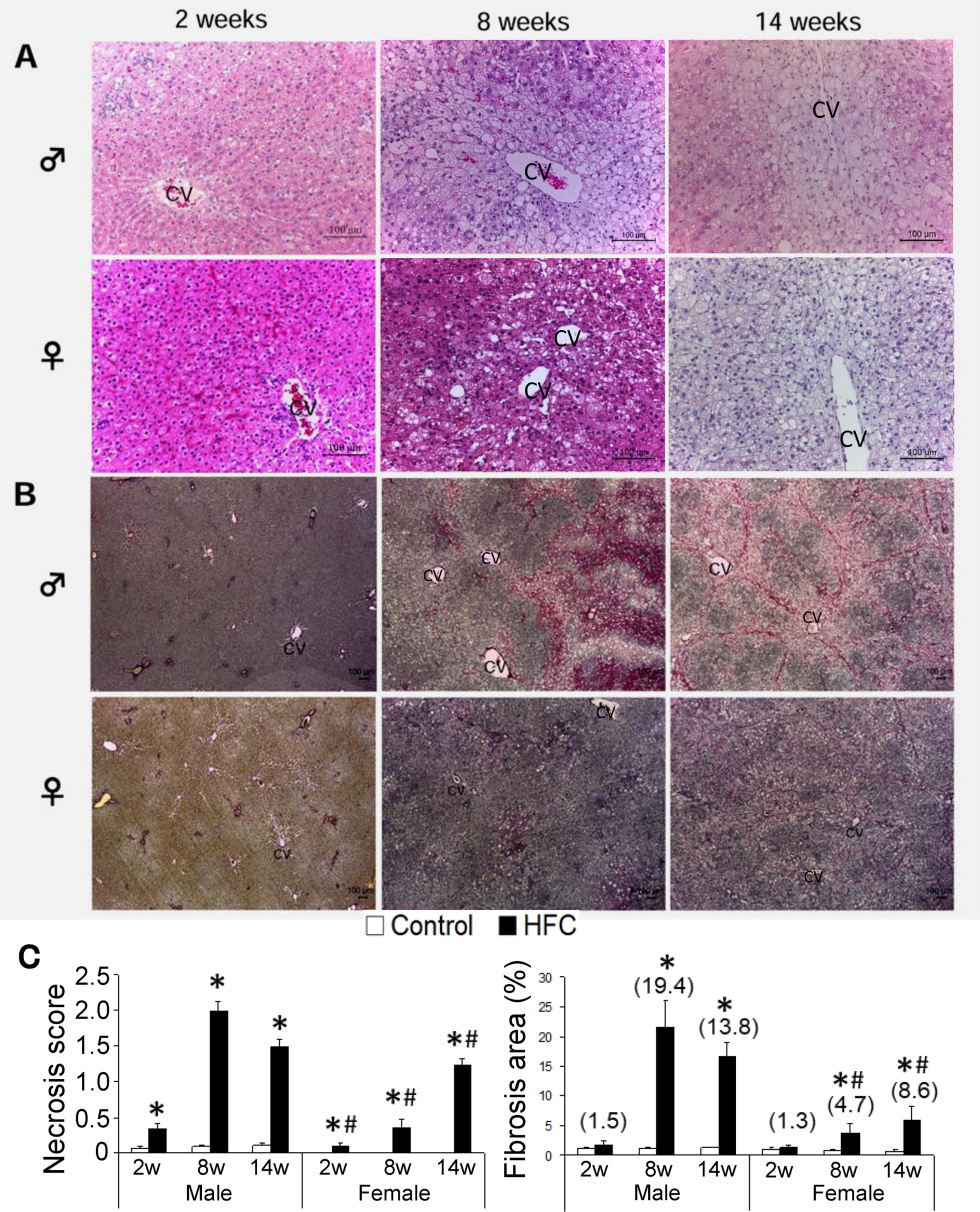
Hepatic H&E and EVG staining, necrosis score, and fibrosis area in rats. Representative liver histology images of male and female rats after 2, 8, and 14 weeks of high-fat-cholesterol (HFC) feeding; (A) hematoxylin and eosin (H&E) staining (magnification ×200); (B) modified Elastic Van Gieson staining using Sirius red (magnification ×40); scale bar, 100 μm. Data are expressed as means ± standard deviations (SD; n = 6/group). Values are presented as fold changes compared with respective controls; CV, central vein; (C) necrosis scores and fibrosis areas (%), n = 6; *P < 0.05 *vs*. control; ^#^*P* < 0.05 female *vs*. male HFC/control ratios.

Histopathological examinations of H&E stained livers showed that the HFC diet induced liver steatosis to a similar degree in male and female rats. Specifically, HFC feeding increased microvesicular steatosis at 2 weeks, and increased macrovesicular steatosis at 8 and 14 weeks (Fig 1A). Perisinusoidal spaces around central veins (CV in Fig 1) were retained after 2-weeks of HFC feeding in male and female rats; however, these spaces were smaller in males than in females and disappeared after 8 and 14 weeks of HFC feeding due to hepatocyte enlargement in both genders. Diet-induced lobular inflammation was almost the same in females as in males, which was a similar phenomenon as observed in the serum markers ALT and AST. The HFC diet also increased hepatocyte ballooning in both genders, but to a significantly smaller degree in females, especially at 14 weeks. Mallory-Denk bodies were occasionally observed in hepatocyte balloons from rats of both genders.

Necrosis and fibrosis were observed in the livers of male and female rats after HFC diet feeding for 8 and 14 weeks, although these were prominently less advanced in female rats than in male rats (Fig 1B and 1C). However, necrosis areas did not increase further in HFC-fed male rats after 14 weeks but did increase further in females. In contrast with assessments of severity, no gender differences were noted in fibrotic regions, and thick fibrotic regions were mainly localized in peripheral areas, especially at the retroperitoneal side after 8 weeks of HFC feeding. At 14 weeks, thick fibrotic regions had faded and honeycomb networks were structured with fibers, and nodules of differing sizes were observed in all microscope fields. Therefore, to further investigate gender differences in the severity of fibrosis, we analyzed indices of fibrogenesis.

### Gender differences in fibrogenic indices

**Fig 2:**
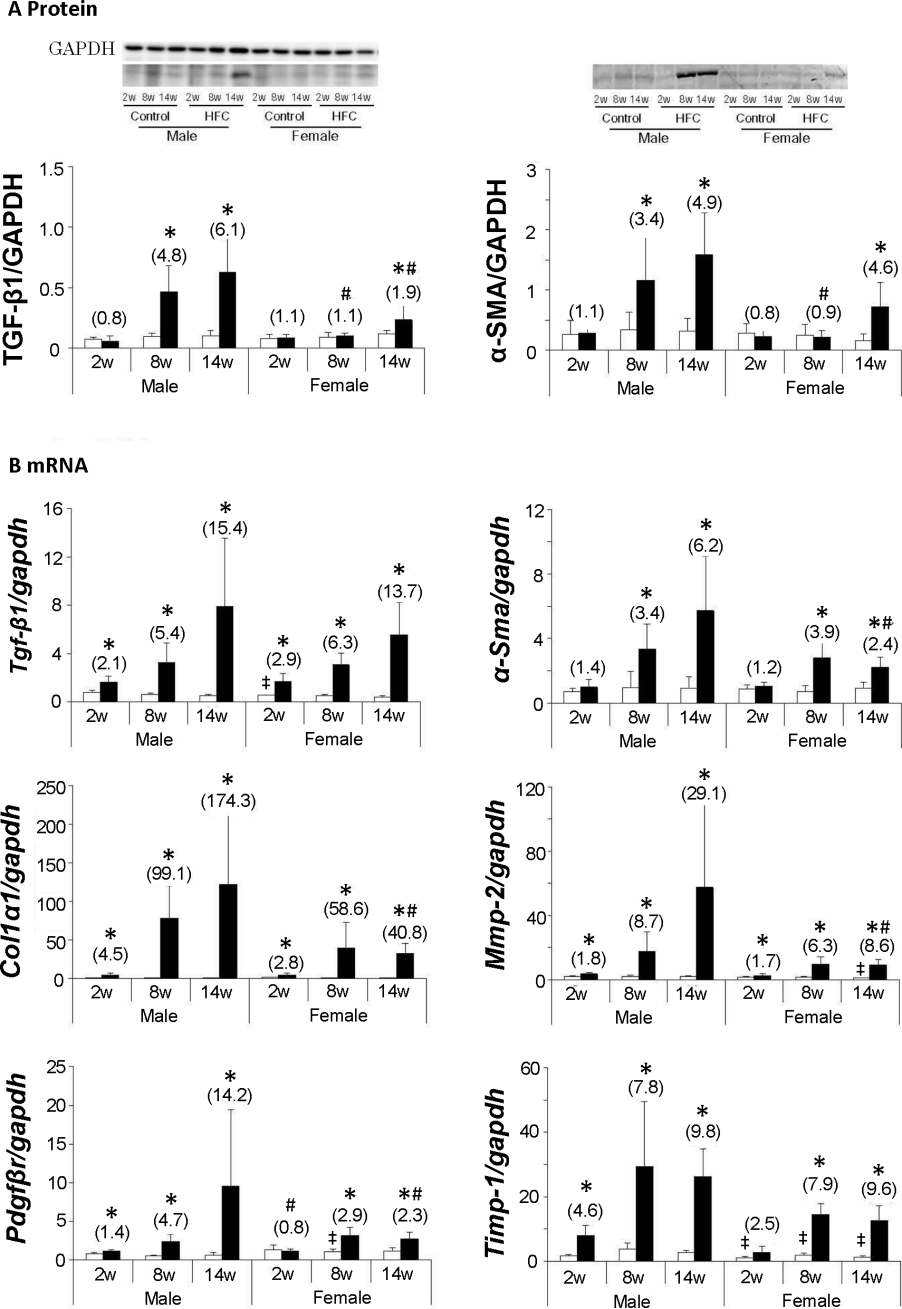
Hepatic fibrosis markers in rats. Representative Western blotting images of the fibrosis-related proteins TGF-β1 and α-SMA (A), and real-time quantitative PCR analyses (B) of genes involved in fibrosis progression, including those encoding TGF-β1 and α-SMA. Data are expressed as means ± SD (n = 6/group). Data in the parentheses are presented as fold changes compared with respective controls. GAPDH was used as a loading control; *P < 0.05 *vs*. control; ^#^*P* < 0.05 female *vs*. male HFC/control ratios; ǂ *P* < 0.05 *vs*. male control. TGF-β1, transforming growth factor β1; α-SMA, alpha smooth muscle actin; GAPDH, glyceraldehyde 3-phosphate dehydrogenase; *Col1α1*, α-1 type I collagen; *Mmp-2*, matrix metallopeptidase-2; *Pdgfβr*, platelet-derived growth factor β receptor; *Timp-1*, tissue inhibitors of metalloproteinases-1.

Previous studies showed that HFC feeding significantly induces the hepatic profibrogenic proteins TGF-β1 and α-SMA in SHRSP5/Dmcr male rats [25]. Herein, the HFC diet increased hepatic TGF-β1 and α-SMA protein expression levels in males at 8 and 14 weeks, but only increased in female rats at 14 weeks (Fig 2A). The increased expression levels were significantly greater in males than in females, except α-SMA at 14 weeks. In accordance, *Tgf-β1* and *α-Sma* mRNA expression was induced by HFC feeding to comparable levels in males and females throughout the study, although fold increases of *α-SMA* mRNA were smaller in female rats than in male rats at 14 weeks (Fig 2B).

Among fibrogenesis related genes, *platelet-derived growth factor-receptor β* (*Pdgfβr*) has been associated with fibrogenesis and with angiogenesis and tumorigenesis [26], Moreover, *α-1 type I collagen* (*Col1α1*) and *matrix metallopeptidase–2* (*Mmp-2*) are reportedly involved in fibrogenesis and the degradation of collagen fibers, respectively, and *tissue inhibitor of metalloproteinase-1* (*Timp-1*) is a known inhibitor of MMPs [27]. Liver *Tgf-β1* and *Timp-1* mRNA constitutive expression levels were greater in male than in female rats. In addition, HFC feeding significantly increased *Col1α1*, *Mmp-2*, *Pdgfβr*, and *Timp-1* mRNA expression in both genders. However, with the exception of *Timp-1* mRNA, these increases were less in female rats than in males, especially at 14 weeks.

### Gender differences in BA kinetic elements

**Fig 3:**
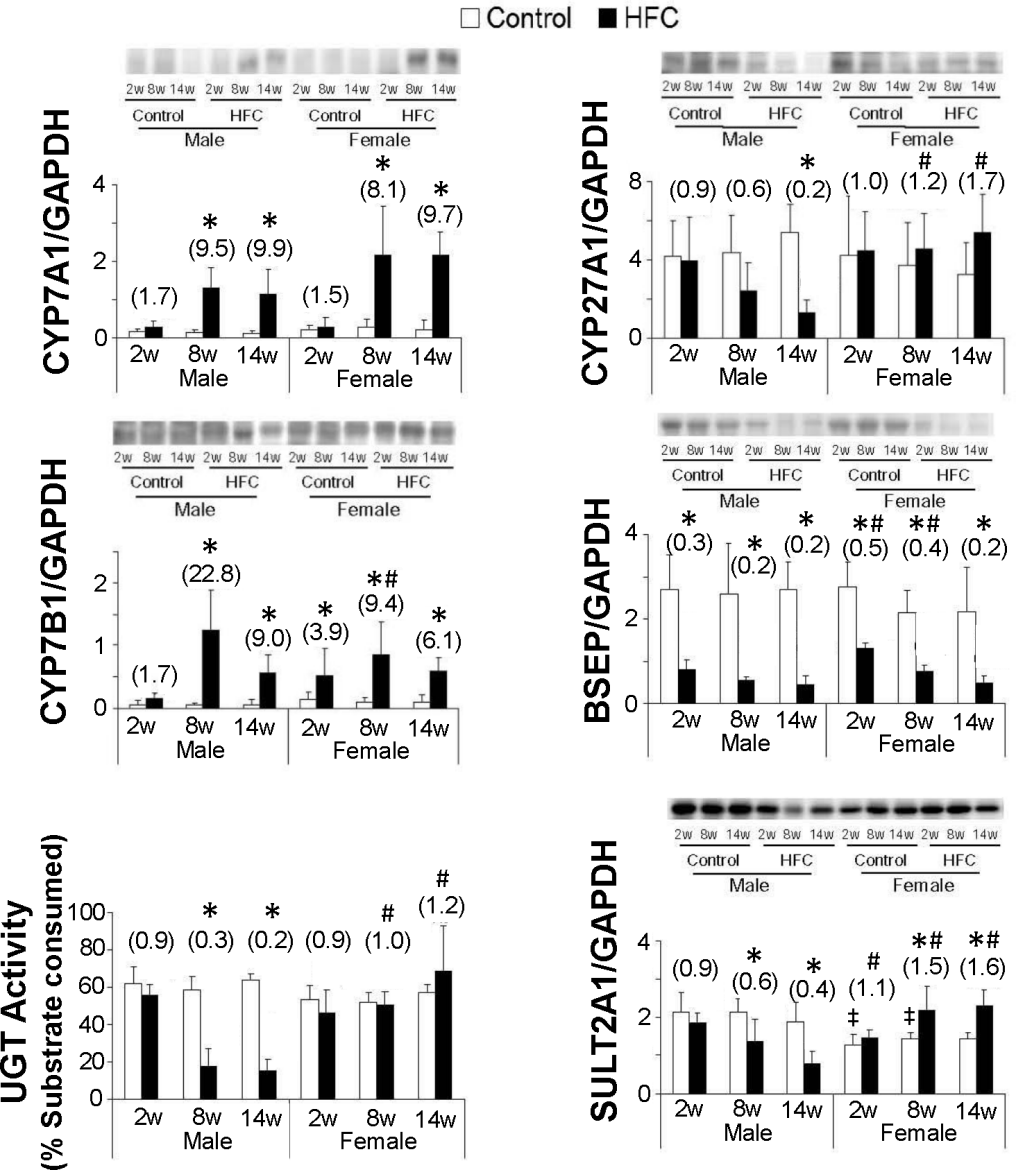
Hepatic BA-related protein markers in rats. Western blot images of proteins involved in BA synthesis, excretion, and detoxification, including CYP7A1, CYP27A1, CYP7B1, BSEP, SULT2A1, and UGT. Substrate consumption (%) by UGT enzymes over 30 min; data are expressed as means ± SD (n = 6/group) and those in the parentheses are presented as fold changes compared with respective controls; *P < 0.05 *vs*. control; ^#^*P* < 0.05, fold differences between HFC and control diets in female and male rats; ǂ *P* < 0.05 *vs*. male control; BSEP, bile salt export pump; CYP7A1, cholesterol 7a-hydroxylase; CYP27A1, sterol 27-hydroxylase; CYP7B1, oxysterol 7a-hydroxylase; HFC, high fat-cholesterol; SULT2A1, sulfotransferase 2A1; UGT, UDP-glucuronosyltransferase.

Previous studies showed that BA synthesis, export, and transformation are dysregulated in HFC-fed male rats [21], and these process have been associated with HFC-induced fibrogenesis [22]. HFC feeding significantly increased CYP7A1 protein levels at 8 and 14 weeks but decreased CYP8B1 (data not shown) protein expression in male and female rats at all time points in comparison with their respective controls (Fig 3). In the alternative BA synthesis pathway, the HFC diet tended to increase CYP27A1 expression in female rats at 8 and 14 weeks, but tended to decrease expression levels of this protein in males at 8 weeks and significantly decreased these at 14 weeks. Hence, the expression of this enzyme was significantly higher in HFC-fed females than in males at 8 and 14 weeks compared with respective controls. In contrast, the HFC diet increased CYP7B1 protein levels in both females and males at all experimental periods except in male rats at 2 weeks, although the ensuing increments were significantly lower in females than in males at 8 weeks. BSEP translocates bile salts into canalicular spaces [28]. Compared with respective controls, HFC feeding suppressed protein expression of BSEP in livers of female and male rats at all time points, but this suppression was greater in male rats at 2 and 8 weeks. Expression levels of the basolateral transporter MRP3 were induced by HFC diet feeding in both males and females (data not shown), whereas UGT activity was unchanged in female rats and was significantly suppressed in males after HFC feeding. Finally, SULT2A1 protein levels were significantly induced by HFC feeding in female rats at 8 and 14 weeks, but were suppressed in male HFC-fed rats.

**Fig 4:**
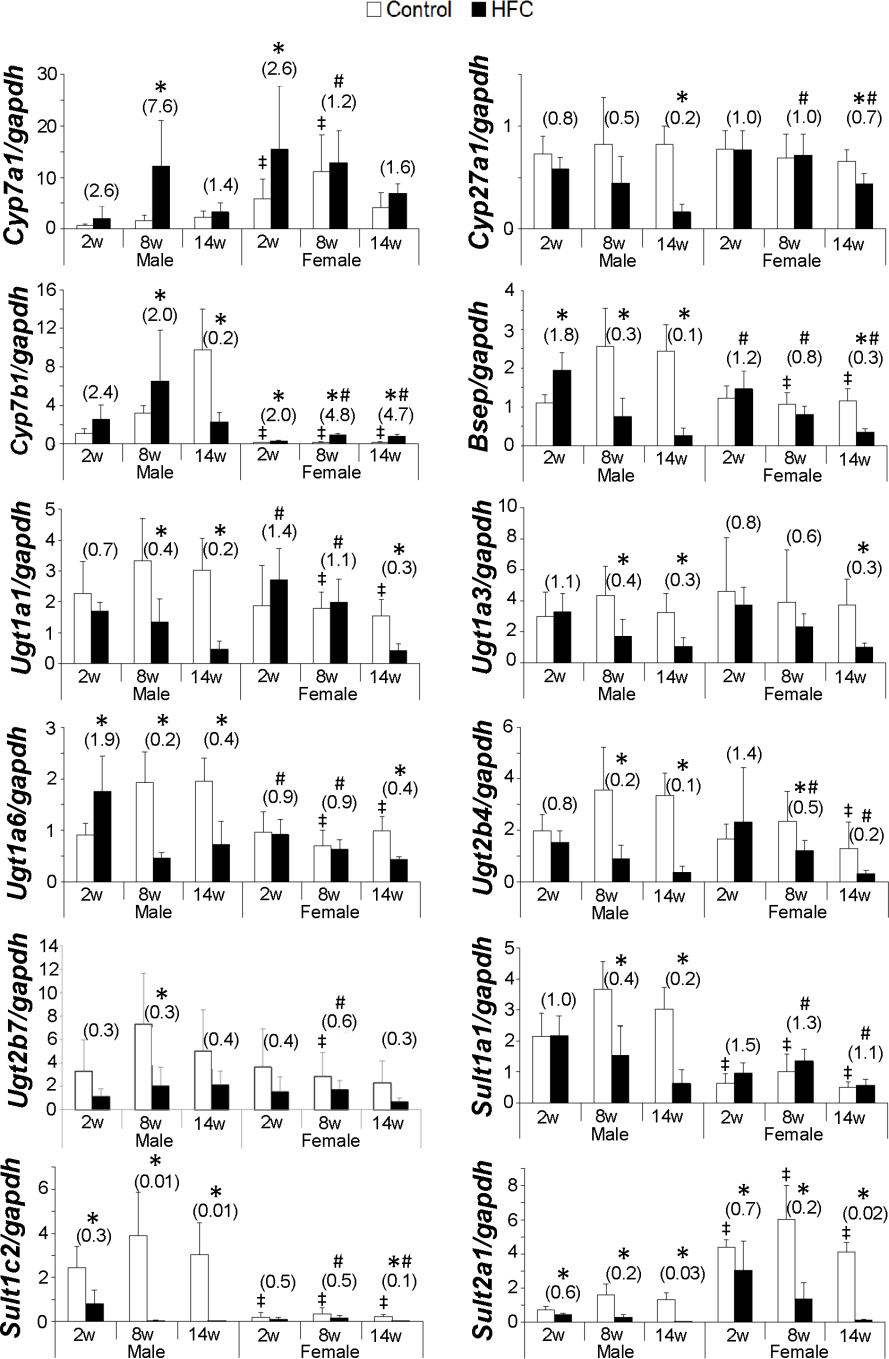
Hepatic BA-related mRNA markers in rats. mRNA expression of molecules involved in BA synthesis, transport, and metabolism, including *Cyp7a1*, *Cyp27a1*, *Cyp7b1*, *Bsep*, and *Sult2a1*; data are expressed as means ± SD (n = 6/group) and those in the parentheses are presented as fold changes compared with respective controls; *P < 0.05 *vs*. Control; ^#^*P* < 0.05 fold changes between female and male; ǂ *P* < 0.05 *vs*. male control.

It was noted that the constitutive expression of hepatic *Cyp7a1* mRNA in female rats at 2 and 8 weeks greater than that of male rats. HFC feeding significantly increased the expression of hepatic *Cyp7a1* mRNA at 2 weeks in female rats and at 8 weeks in males compared with respective controls, and the fold increase in males was significantly greater than that in females, regardless of no sex difference in the protein elevations (Fig 4). This discrepancy in the effect of HFC diet between *Cyp7a1* mRNA and the protein was also reported earlier [18]. However, HFC feeding suppressed hepatic *Cyp8b1* mRNA levels in rats of both sexes at all time points, and decreases in *Cyp8b1* levels were much lower in female than in male rats at 8 and 14 weeks (data not shown). *Cyp27a1* mRNA levels were significantly suppressed by HFC feeding in male and female rats at 14 weeks. However, this suppression was less pronounced in female than in male rats at 14 weeks. Moreover, HFC-fed female rats had higher *Cyp7b1* mRNA levels than control diet-fed rats throughout the study. In contrast, male HFC-fed rats had lower mRNA levels than corresponding controls at 14 weeks, and significantly higher *Cyp7b1* mRNA expression levels than females at 8 and 14 weeks. Expression levels of *Bsep* mRNA were significantly increased in female HFC-fed rats at 2 weeks, but were significantly decreased in males at 8 and 14 weeks, and to a lesser extent in females at 14 weeks. Finally, HFC diet-mediated increases in *Mrp3* mRNA levels were significantly greater in females than males at 8 weeks (data not shown).

In mRNA expression analyses of *Ugt* and *Sult isoforms* in the liver, no gender differences were identified among control animals, whereas expression levels of *Sult1a1*, *1c2*, and *2a1* mRNAs differed significantly between males and females. Specifically, female rats had greater expression of *Sult2a1* than male rats, and the opposite differences were observed for the other two *Sult isoforms*. HFC feeding time-dependently suppressed *Ugt1a1*, *1a3*, *1a6*, and *2b4* mRNA levels in male and female rats, although degrees of suppression were significantly less in females than in males, except for that of *isoform 1a3*. HFC feeding inhibited *Sult2a1* and *1c2* mRNAs throughout the study in both gender groups, but decreases in female rats were not as pronounced as in male rats. HFC feeding also suppressed *Sult1a1* mRNA expression in male rats, but did not have this effect in females at any of the tested time points. In contrast, HFC-mediated decreases in liver *Sult1c2* and *2a1* expression levels were less in females than in males.

### Gender differences in nuclear receptor expression

**Fig 5:**
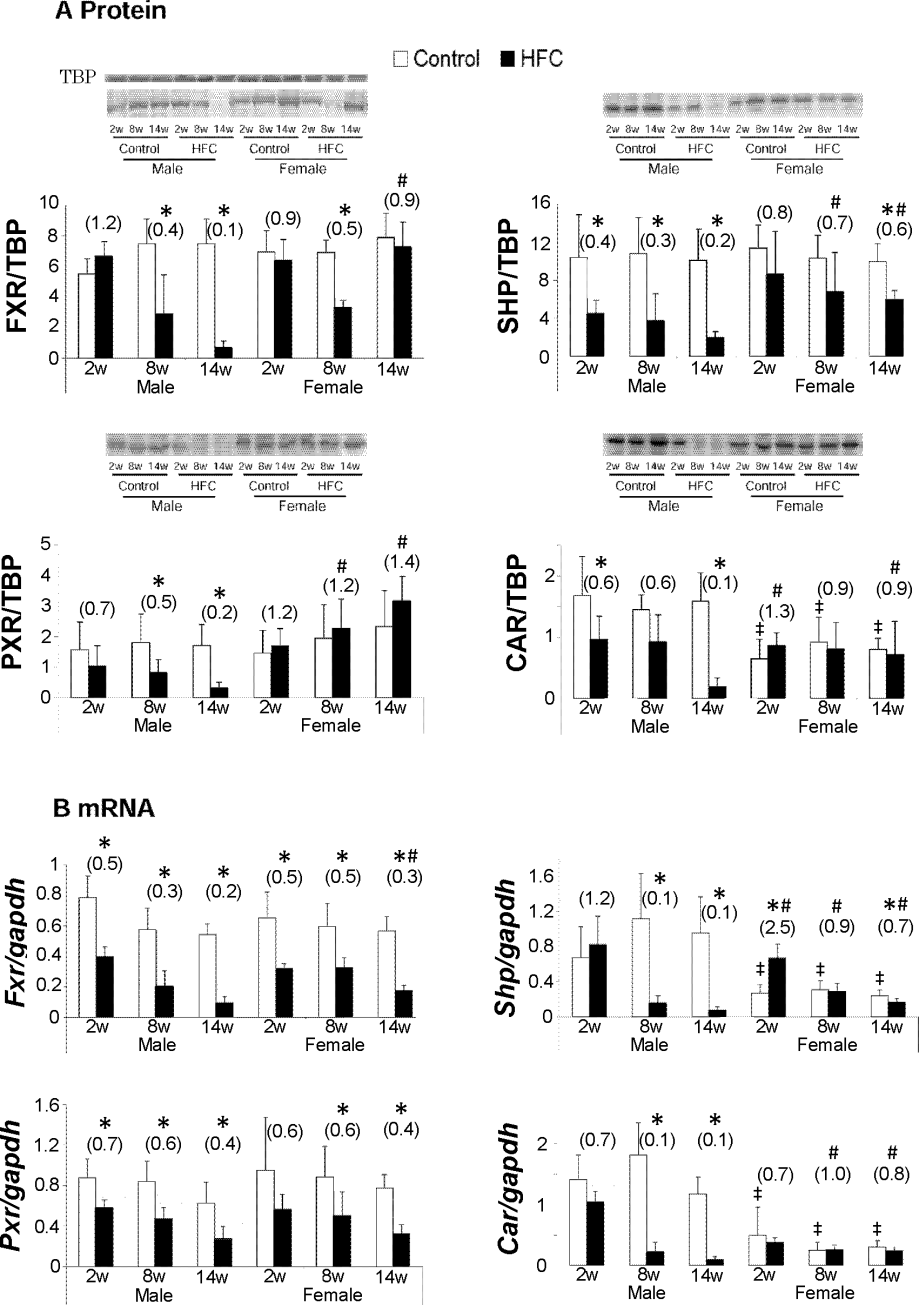
Hepatic nuclear receptors in rats. Nuclear regulators of BA homeostasis; (A) Hepatic protein levels of FXR and its target genes encoding SHP, PXR, and CAR were determined using Western blotting. (B) Real-time quantitative PCR of *Fxr*, *Shp*, *Pxr*, and *Car* mRNA; data are expressed as means ± SD (n = 6/group). Data in the parentheses are presented as fold changes compared with respective controls. TBP was used as a loading control in nuclear fraction; ^#^*P* < 0.05 female *vs*. male HFC/control ratios; ǂ *P* < 0.05 *vs*. male control. CAR, constitutive androstane receptor; FXR, farnesoid X receptor; PXR, pregnane X receptor; SHP, small heterodimer partner; TBP, TATA binding protein.

Nuclear receptors play pivotal regulatory roles in hepatic lipid metabolism and contribute to BA homeostasis, and to the development of steatosis and inflammation [29]. Sexual dimorphisms of nuclear receptors have been shown in previous studies [30]. Thus, we determined expression levels of nuclear receptors that regulate bile acid synthesis and detoxification in the present groups of animals. HFC feeding significantly downregulated FXR protein expression in male rats at 8 and 14 weeks, but it decreased in female rats only at 8 weeks (Fig 5A). At the mRNA level, HFC feeding downregulated *Fxr* expression in both male and female rats, although these decreases were less in females than in males (Fig 5B). HFC-diet feeding significantly downregulated protein expression of the FXR target SHP at 14 weeks in females and at all time points in male rats, and these decreases were significantly less in females than males. Moreover, HFC feeding slightly increased *Shp* mRNA levels in male rats at 2 weeks and significantly decreased them thereafter. In contrast, HFC feeding significantly increased *Shp* mRNA expression in female rats at 2 weeks. PXR protein expression levels were significantly downregulated in male HFC-fed rats at 8 and 14 weeks, but tended to be upregulated in HFC-fed females at all time points. However, the HFC diet suppressed *Pxr* mRNA levels in both male and female rats throughout the study. Compared with control rats, HFC feeding tended to increase CAR protein levels in female rats at 2 weeks and did not influence these thereafter, whereas CAR protein expression was significantly decreased in livers of male HFC-fed rats at 2 and 14 weeks. Accordingly, *Car* mRNA levels were similar between control and HFC-fed female rats but were significantly lower in HFC-fed male rats than in control male rats at 8 and 14 weeks.

## Discussion

In our previous studies of the pathogenetic mechanisms behind fibrotic steatohepatitis in male SHRSP5/Dmcr rats we showed that HFC feeding leads to cholesterol accumulation in the liver. Consequent alterations in BA metabolism and liver accumulation of toxic BAs such as chenodeoxycholate (CDCA) [19, 22] contributed to HFC diet-induced liver damage and especially fibrosis [22]. The present histological and biological analyses indicated that HFC feeding also induces fibrotic steatohepatitis in SHRSP5/Dmcr female rats. However, female rats were clearly less susceptible to HFC diet-induced liver injury compared with males, especially in terms of fibrotic changes. These observations were supported by changes in the expression of the fibrogenesis markers TGF-β1, α-SMA, *Col1α1*, and *Mmp-2*. Hence, the present gender differences may reflect differing responses of drug-metabolizing enzymes (UGT and SULT) that detoxify toxic BAs, and their nuclear receptors (PXR and CAR). Similarly, our data suggest that FXR and SHP are important contributors to gender differences.

The prevalence of NAFLD is higher in men than in premenopausal women, and increases in women immediately after menopause [31–33]. Hence, estradiol is likely protective against the progression of human NAFLD/NASH. In this study, we subjected 10-week-old female rats to a HFC-diet and monitored the resulting liver damage from 12 to 24-weeks of age. Rats are mature at these ages, which correspond with menopausal ages in humans. Therefore, HFC-fed SHRSP5/Dmcr rats closely resemble NASH/NAFLD and offer a useful animal model for studies of gender differences in HFC-induced liver damage. HFC-diet feeding significantly decreased serum estradiol levels in female rats at only 2 weeks; however, the levels in HFC-fed female rats were still over 100-fold higher compared with those of adult control males [34], indicating that this sex hormone is a pivotal mediator of gender differences in HFC-induced fibrotic steatohepatitis.

In addition to differences in levels of the female sex hormone, nuclear receptor-regulated liver metabolic pathways for detoxification of toxic BAs, BA synthesis [35], and hepatic regulation of cholesterol homeostasis [36] are reported contributors to sexual differences [30]. In general, nuclear receptors play pivotal roles in gender-specific metabolic pathways, including those that influence liver function in animal models of health and disease. Accordingly, female mammals are less vulnerable to liver injury due to comparatively robust nuclear receptor networks [30]. Hence, sex differences in fibrogenesis of HFC diet-fed SHRSP5/Dmcr rats might be associated with sensitivities of detoxification enzymes in the liver. In particular, mRNA levels of the *UGTs Ugt1a1*, *Ugt1a6*, and *Ugt2b4*, SULT2A1 protein and mRNA, and *Sult1a1* and *1c2* mRNA levels, were resistant to the effects of the HFC diet in female SHRSP5/Dmcr rats compared with those in male rats. Similarly, the nuclear receptors CAR and PXR were regulated in a similar fashion. Hence, as shown in SHRSP and SHESP5/Dmcr male rats [18], these data suggest the importance of phase II reactions of drug-metabolizing enzymes in NASH/NAFLD progression and differing sensitivities to HFC feeding. UGT and SULT are involved in detoxification of toxic BAs such as CDCA and glyco-formed BAs [37, 38], and whereas *Ugt1a1* contributes to bilirubin metabolism, *Ugt1a3*, *Ugt2b4*, and *Ugt2b7* metabolize BA (24-OH), BA (6-OH), and BA (3-OH), respectively, [39–41]. With the exceptions of *Ugt1a3* and *2b7*, the present HFC diet had smaller effects on *UGT isoforms* in female rats at early stages, and although the roles of *Ugt1a6* in BA metabolism remain unknown, this isoform was relatively unaffected by HFC feeding in females. CAR and PXR protein expression levels were resistant to HFC feeding in female SHRSP5/Dmcr rats, whereas time-dependent decreases were observed in male HFC-fed rats. Consistent with the influence of these receptors on target *Sult* genes, HFC feeding significantly induced the expression of SULT2A1 protein, despite significantly suppressing mRNA expression levels. HFC feeding also decreased both mRNA and protein expression levels of SULT2A1 in male rats, and had smaller effects on *Sult1a1* and *1c2* mRNA levels in females than in males. These data suggest that estradiol stabilizes CAR and PXR proteins and the ensuing expression levels of the target genes for UGT and SULT [30], which accelerate toxic bile acid detoxification and protect against hepatic fibrogenesis. This means that HFC feeding comprised the redox function of hepatocytes in males, but had no similar effect in females. Accordingly, HFC-induced fibrogenesis markers *Col1α1* and *Pdgfβr* were transcribed at lower levels in females than in males. Collectively, these observations suggest that as estradiol levels decrease in women after menopause, and the prevalence of NASH/NAFLD suddenly increases due to cessation of estradiol-mediated protective effects. In agreement, estradiol was reportedly protective against CCl_4_-induced liver fibrosis in rats [42]. However, further studies are required to clarify the related mechanisms.

Alnouti *et al*. [15] showed that gender-specific SULT2A1 activity and expression reflected stimulatory effects of estrogens and suppressive effects of androgens and male growth hormones. Similarly, *Sult2a1* mRNA levels were at least 6-fold higher in female livers than in male livers [43, 44], and were 4-fold higher in female than male SHRSP5/Dmcr rats, although no differences in the protein expression were observed. In contrast, *Sult1a1* and *1c2* expression levels were higher in male than female SHRSP5/Dmcr rats, suggesting that gender differences in SULT expression vary between isoforms. It is well known that UGT and SULT are regulated by CAR and PXR, and constitutive expression of CAR protein and mRNA were lower in livers from female SHRSP5/Dmcr rats than in their male counterparts. However, no gender differences in PXR protein and gene expressions levels were observed, although hepatic CAR levels were reportedly higher in women than men [45]. Therefore, the available expression data for these two nuclear receptors and estrogen are insufficient to fully explain the constitutive expression of SULT isoforms, and further investigations are needed.

BAs are produced via catalytic actions of CYP enzymes such as CYP7A1, CYP27A1, CYP8B1, and CYP7B1 [22], and are detoxified by the protein products of UGT1A3, UGT2B7, UGT2B4 [40], and SULT2A1 [46]. No gender differences were observed in the expression of BA producing enzymes except for CYP27A1 and CYP7B1, or in expression levels of cellular BA exporters such as MRP3 after HFC-diet feeding. However, lower suppression of CYP27A1 and BSEP, and higher induction of CYP7B1 were observed in females compared with males, suggesting that HFC-diet feeding produces higher concentrations of BAs in females than in males, whereas excretion and detoxification activities are stronger in females. These kinetic differences may reflect gender differences in the accumulation of toxic BA, with greater BA accumulation in males than in females. Accordingly, gender differences in BA synthesis might contribute to NASH development, and to fibrogenesis in HFC diet-fed SHRSP5/Dmcr rats.

Physiologically, no gender differences in serum and hepatic TC are likely in adult rats, although TC levels were reportedly limited by 3-hydroxy 3-methylgultaryl coenzyme A reductase in livers of females [36]. Despite the reported relationship between this enzyme and estrogen expression levels, no gender differences in TC or TG levels were observed in serum and liver samples from SHRSP5/Dmcr rats fed the control diet. However, gender differences in serum and hepatic TC levels and serum TG levels were significant at all observed points except at 14 weeks in serum TC levels among HFC-fed rats. HFC diet-feeding increased serum TC levels more in female rats than in male rats, and increased liver TC levels more in female than in male HFC-fed rats. Because excess cholesterol is ultimately catabolized to BAs in the livers of female SHRSP5/Dmcr, lower liver TC levels were expected in females. However, serum TC levels were significantly higher in HFC diet-fed female rats than in male rats. Moreover, HFC feeding increased serum TG levels more in female rats than in male rats, although no gender differences were observed in liver TG responses to HFC feeding. Thus, future studies are required to investigate lipid homeostasis after HFC-diet feeding to further characterize the effects on pathways of TC and TG homeostasis.

FXR is highly expressed in the liver, and CDCA and other BAs are natural ligands for this receptor [47]. One of the primarily roles of FXR activation is to downregulate CYP7A1 via SHP, and subsequently to moderate BA accumulation [48]. In agreement, FXR activation suppressed the development of liver fibrosis [49]. Moreover, in the present male rats, FXR and SHP were downregulated by HFC feeding, and induced CYP7A1. In contrast, these receptors were not downregulated in female HFC-fed rats, and CYP7A1 was adversely induced. Few published studies report sexually dimorphic sensitivities of FXR and SHR expression to HFC feeding. However, the present experiments showed reduced sensitivity of mRNA and protein expression levels to HFC-diet feeding in female rats compared with male rats. In contrast, CYP7A1 protein expression was not dimorphic, although *Cyp7a1* mRNA induction was lower in female than in male rats after 8 weeks of HFC-diet feeding. These observations warrant further studies to determine whether estrogen influences *Fxr* and *Shr* mRNA and protein expressions. Swanson *et al*. [50] showed that the intestinal FXR/FGF15 pathway was critical for suppressing the expression of *Cyp7a1* and *Cyp8b1* genes, whereas the liver FXR/SHP pathway was important for suppressing *Cyp8b1* gene expression and played a minor role in suppressing *Cyp7a1* gene expression in mice. Because the influences of HFC-diet feeding on the expression of both *Cyp* genes are dimorphic, investigations of the intestinal FXR/FGF15 pathway may provide clear indications of gender differences.

Considering the increasing risk of NASH in women after menopause, higher levels of serum feritin might be involved, because the levels were reported to be higher in women before menopause [51]. Elevations in serum ferritin and iron are common in NASH [52], which induced hepatic inflammations due to oxidative stress [53]. However, we could not investigate the iron metabolism as well as HFC affect on female rats after menopuse in this study, further investigations are warranted.

## Conclusion

In conclusion, similar to gender differences in human NASH/NAFLD, HFC-diet feeding gender-dependently induced liver damage in SHRSP/5Dmcr rats. The present data suggest that comparative female resistance to liver damage and fibrosis reflects stronger detoxification of toxic endogenous substances derived from HFC-diet feeding. Specifically, the hepatic receptors CAR and PXR and their target BA detoxification genes *Ugt* and *Sult* were slightly decreased in female HFC-fed rats, but were strongly decreased in male rats under these conditions.

## Supporting information

S1 Table List of primer sequences for real-time quantitative PCR. Gapdh, glyceraldehyde 3-phoshate dehydrogenase; Tgf-β1, transforming growth factor-β1; αSma, α-smooth muscle actin; Col1a1, α-1 type I collagen; Mmp2, matrix metallopeptidase-2; Pdgfβr, platelet-derived growth factor receptor β; Timp1, tissue inhibitor of metalloproteinase -1; Cyp, cytochrome P450 enzymes; Bsep, bile salt export pump; Ugt, UDP-glucuronosyltransferase; Sult, sulfotransferase; Fxr, farnesoid X receptor; Shp, small heterodimer partner; Pxr, pregnane X receptor; Car, constitutive androstane receptor.
(DOCX)
