# Supplementary material for: Bile acid detoxifying enzymes limit susceptibility to liver fibrosis in female SHRSP5/Dmcr rats fed with a high-fat-cholesterol diet
Source: PLoS One. 2018 Feb 13;13(2):e0192863. doi: 10.1371/journal.pone.0192863 (PMC5811017; doi:10.1371/journal.pone.0192863)
Supplement: S1 Table — Gapdh, glyceraldehyde 3-phoshate dehydrogenase; Tgf-β1, transforming growth factor-β1; αSma, α-smooth muscle actin; Col1a1, α-1 type I collagen; Mmp2, matrix metallopeptidase-2; Pdgfβr, platelet-derived growth factor receptor β; Timp1, tissue inhibitor of metalloproteinase -1; Cyp, cytochrome P450 enzymes; Bsep, bile salt export pump; Ugt, UDP-glucuronosyltransferase; Sult, sulfotransferase; Fxr, farnesoid X receptor; Shp, small heterodimer partner; Pxr, pregnane X receptor; Car, constitutive androstane receptor. (DOCX) [file pone.0192863.s001.docx]

Supplemental Table 1. List of primer sequences for real-time quantitative PCR

| Genes | Number | Forward (5′–3′) | Reverse (5′–3′) |
| --- | --- | --- | --- |
| *Gapdh* | BC096440 | AGAACATCATCCCTGCATCCA | CCGTTCAGCTCTGGGATGAC |
| *Tgfb1* | NM_021578 | CAACAATTCCTGGCGTTACCTT | GACGTCAAAAGACAGCCACTCA |
| *αSma* | NM_031004 | ATGGGCCAAAAGGACAGCTA | TGATGATGCCGTGTTCTATCG |
| *Col1a1* | NM_053304 | ATGCTTGATCTGTATCTGCCACAAT | ACTCGCCCTCCCGTTTTT |
| *Mmp2* | 146262018 | TGAGCTCCCGGAAAAGATTG | CATTCCCTGCGAAGAACACA |
| *PdgfβR* | NM_031525 | GCACCGAAACAAACACACCTT | ATGTAACCACCGTCGCTCTC |
| *Timp1* | NM_053819 | TACCAGAGCGATCACTTTGCCT | GAGACCCCAAGGTATTGCCAG |
| *Cyp7a1* | NM_012942 | AGCTGGCTGAGGGATTGAAG | GAATAGCGAGGTGCGTCTTG |
| *Cyp7b1* | NM_019138 | TAGGACTAAACCACAGTCGC | TGCAGCCTTATTCCGCTA |
| *Cyp27a1* | M73231 | CCTTTGGGACTCGCACCA | GCCCTCCTGTCTCATCACTTG |
| *Bsep* | NM_031760 | GCCATTGTGCGAGATCCTAAA | TGCAGGTCCGACCCTCTCT |
| *Ugt1a1* | NM_012683 | ACACAGATCGCATGAACTTCCTG | AGGACTCAGAAGGTCCTTGACAGTC |
| *Ugt1a3* | NM_201424 | AGGGGTCTGCTGCATAACAC | AGGAACACGCAGGTACATGG |
| *Ugt1a6* | AF461737 | GCTATCGCTCCTTTGGGAACA | CCTTCAGGAGGCTCTGGCAG |
| *Ugt2b4 (Ugt2b35)* | NM_001004271 | TCTCAGATGCCATTGGTCCC | GTGTAGCCAGGAGAGAAGCG |
| *Ugt2b7* | NM_173323 | AGATGCTGTTGGTCCCTGTG | AAGAGGGAGGGAAGGGTTGT |
| *Sult1a1* | NM_031834 | TACTGGGAACCAAGGCAAAC | GCGTTGATAAAACCCTCTGC |
| *Sult1c2* | NM_133547 | AGAGCAGAATGGAGGCTTGA | AGTGGGAGGTGGAAGGAAGT |
| *Sult2a1* | NM_131903 | CCAGATGAGCTGGATTTGGT | CCCAATAGTGCCTTTCCTCA |
| *Fxr* | NM_021745 | CAGTGAATGAGGACAGCGAA | CATAGCTTGGTCGTGGAGGT |
| *Shp* | NM_057133 | CCTTGGATGTCCTAGGCAAG | CCAACCCAAGCAGGAAGAG |
| *Pxr* | AF151377 | ATCTCCCTCTTCTCCCCAGA | TACCACGCTACGTTGAACCA |
| *Car* | AB104736 | ACAAGATGGAAGATGCGGTC | GGGTTTTGTGGAAGTGGATG |
